# The Impact of Pharmacist-Led Medication Management Services on the Quality of Life and Adverse Drug Reaction Occurrence

**DOI:** 10.3390/pharmacy10050102

**Published:** 2022-08-25

**Authors:** Andrea Brajković, Lucija Ana Bićanić, Marija Strgačić, Helena Orehovački, Djenane Ramalho-de-Oliveira, Iva Mucalo

**Affiliations:** 1 Centre for Applied Pharmacy, Faculty of Pharmacy and Biochemistry, University of Zagreb, 10000 Zagreb, Croatia; 2 City Pharmacies Zagreb, 10000 Zagreb, Croatia; 3 Centre for Pharmaceutical Care Studies, College of Pharmacy, Federal University of Minas Gerais, Belo Horizonte 31270-901, MG, Brazil

**Keywords:** health-related quality of life, adverse drug reactions, comprehensive medication management, pharmacist, cardiovascular diseases, older patients

## Abstract

The aim of this research was to assess the impact of comprehensive medication management (CMM) services on patients’ health-related quality of life (HRQoL) and frequency of adverse drug reactions (ADRs) in older patients with cardiovascular diseases (CVDs). A prospective, pre- and post-intervention study with a one-year follow-up was conducted at the Health Care Centre Zagreb—Centre (HCZC). The Euro-Quality of Life Questionnaire 5 Dimensions 5 Levels (EQ-5D-5L) was used to measure the HRQoL at baseline (initial visit at the HCZC) and 12 months following CMM services. The ADRs collected at the initial assessment of the CMM services and throughout follow-up consultations were analyzed according to the occurrence mechanism, seriousness, expectedness and distribution of the Preferred Term according to the System Organ Class. Following the CMM intervention, 65 patients reported significant improvement in dimensions “self-care” (*p* = 0.011) and “usual activities” (*p* = 0.003), whereas no significant change was found in the “mobility” (*p* = 0.203), “pain/discomfort” (*p* = 0.173) and “anxiety/depression” (*p* = 0.083) dimensions and the self-rated VAS scale (*p* = 0.781). A total of 596 suspected ADR reports were found, the majority at patients’ initial assessment (67.3%), with a mean ± SD of 9.2 ± 16.9 per patient. The CMM services significantly reduced the rate of suspected ADRs, namely 2.7 ± 1.7 ADRs per patient at the initial assessment vs. 1.0 ± 1.5 ADRs per patient at the last consultation (*p* < 0.001). The obtained results indicate that CMM services may improve patients’ HRQoL. Additionally, as CMM services diminished the proportion of ADRs following 1-year patient follow-up, they may serve as a viable solution for safety management.

## 1. Introduction

Since cardiovascular diseases (CVDs) present the leading comorbidity and cause of death in Croatia often requiring long-term and complex medication use, patients with CVDs are at a higher risk of having less effective treatment, a higher prevalence of adverse drug reactions (ADRs) and increased health care utilization [1]. Studies have shown that these patients have a reduced health-related quality of life (HRQoL), worse clinical outcomes and present a significant financial burden to the health care system [2,3,4,5,6,7,8]. Therefore, medication management is deemed crucial for the treatment of CVDs and their modifiable risk factors, and pharmaceutical care practice has emerged as a solution to the abovementioned predicaments [9,10].

Identifiable events in pharmaceutical care practice are termed comprehensive medication management services (CMM services), and they present an evidence-based and patient-centered service where a pharmacist is held responsible for patients’ drug-related needs and accountable for this commitment. Pharmacists use the theoretical framework proposed by Cipolle et al. to prevent, identify and resolve drug therapy problems, develop a care plan and provide continuous follow-up to achieve positive clinical outcomes, reduce unwanted adverse effects and improve patients’ quality of life [9,11]. Along with the improvement of clinical and economic outcomes, HRQoL is considered a fundamental objective of the provision of pharmaceutical care practice.

HRQoL represents a multidimensional assessment of a patient’s physical, functional, psychological and social health [12]. Insights into the patients’ HRQoL provide new findings on the impact chronic diseases have on life, and in the past few years, HRQoL became an important indicator of therapeutic benefit and health outcomes in patients with CVDs [2,3]. Even more, since the COVID-19 pandemic started, the HRQoL has further been adversely affected by its detrimental effect [13,14]. Regardless of the growing body of evidence, disparate studies that have explored the influence of a multitude of pharmacy interventions on patients’ HRQoL have found diverse results [15]. To the best of the authors’ knowledge, no study to date has examined the impact of pharmaceutical care practice that follows the theoretical framework proposed by Cipolle et al., namely CMM services, on patients’ HRQoL at the primary care level. Hence, the primary objective of this research was to assess the impact of CMM services on the HRQoL before and during the COVID-19 pandemic in older patients with CVDs at the county health center in Croatia.

Furthermore, it has been noticed that when drug therapies produce adverse effects, studies mainly focus on their clinical and physical impact (namely whether they resulted in death, life-threatening conditions, inpatient hospitalization, prolongation of existing hospitalization, persistent or significant disability/incapacity, etc.) rather than on the evaluation of all the aspects of a patient’s HRQoL. Despite the growing interest in HRQoL, there is not much information about the quality of life among patients with ADRs [16]. Previous studies have shown that ADRs have an unfavorable impact on patients’ HRQoL [16,17,18,19], and it is the elderly patients with multiple chronic comorbidities and polypharmacy who are at a significantly increased risk of experiencing ADRs [20,21,22,23]. In addition, according to the authors, this is the first research with an in-depth analysis of ADRs in patients receiving CMM services. Therefore, the secondary aim of the present study was to evaluate the impact of CMM services on the frequency of ADRs in cardiovascular patients and to ascertain their extent and type.

## 2. Materials and Methods

### 2.1. Study Design and Setting

This prospective, pre- and post-intervention study with a one-year follow-up was conducted from January 2018 to December 2020 at the Health Care Centre Zagreb—Centre (HCZC). Presented data represent a secondary subset analysis of trial data evaluating the clinical impact produced by CMM services in patients with hypertension and at least one additional established CVD as a primary outcome measure [24]. The CMM services provided at the HCZC were developed in cooperation with the University of Zagreb (UoZ) Faculty of Pharmacy and Biochemistry whose staff was in charge of the implementation and provision of the CMM services. The detailed process of pre- and early implementation of this novel practice management system of CMM services was presented elsewhere [25].

### 2.2. Study Participants and Data Collection

Study participants aged 65 to 80 years, with diagnosed hypertension and at least one additional established CVD were enrolled in the study. Exclusion criteria included mental and behavioral disorders due to psychoactive substance use, behavioral syndromes, cognitive impairment and inability to decide independently on health-related aspects. Patients eligible for the study were identified based on the pre-defined inclusion criteria by their general practitioners and/or medical specialists and then referred to pharmacists. All the anthropometric, sociodemographic and clinical data were collected by a review of patients’ medical records and the interview during the initial consultation at the HCZC. Patients’ HRQoL, the primary outcome of the study, was measured by the Euro-Quality of Life Questionnaire 5 Dimensions 5 Levels (EQ-5D-5L) at baseline (initial visit at the HCZC) and 12 months following pharmacists’ intervention (CMM services).

### 2.3. Health-Related Quality of Life Assessment Tool

The EQ-5D-5L is a questionnaire that consists of an EQ-5D descriptive system with five dimensions measuring mobility, self-care, usual activities, pain/discomfort and anxiety/depression and an EQ visual analog scale (EQ VAS) measuring patients’ overall current health. For the purposes of this study, the questionnaire translated into the Croatian language and validated in the Croatian version was used [26]. EQ-5D-5L health state was represented by a 5-digit code, which states a unique health state for each individual. There are 3125 possible health states defined. The impact of CMM services on HRQoL was assessed by the changes in the distribution of responses to the self-care and usual activities dimensions of the EQ-5D-5L. For the purpose of detecting change in health status over time, an EQ-5D health state was assumed to be “better” than another if it was better on at least one dimension and no worse in any other dimension [27]. The participants completed questionnaires with the assistance of pharmacists-researchers providing the service.

### 2.4. Pharmacy Intervention

Pharmacists providing CMM services followed the validated standardized process used to assess initial information, identify, resolve and prevent drug therapy problems (DTP), develop a patient care plan and reassess new information, which is followed up with the patients’ health status [9]. In doing so, pharmacists also determined personalized therapy goals, chose interventions and evaluated outcomes, all to achieve the best feasible health status and reach the highest possible quality of life. The workflow included collaboration with both general practitioners and patients to implement suggested interventions and provide care at the highest possible level.

### 2.5. Adverse Drug Reactions

Case reports collected at the initial assessment of the CMM services and throughout follow-up consultations were stored in the CMM documentation system and used as the data source. Data concerning ADRs that were experienced prior to approaching and during CMM services and were possibly, probably or certainly related to the use of the suspected drug [28,29,30] were taken into account. Once identified, suspected ADRs were coded into the related Preferred Term (PT) using the Medical Dictionary for Regulatory Activities (MedDRA) terminology [31] and further analyzed with respect to the total and average number of reports per patient, sequence number of consultation, baseline characteristics of patients (including age, sex, number of drugs used and number of comorbidities), distribution of PT according to the System Organ Class (according to MedDRA [31]), occurrence mechanism (according to Edwards and Aronson [28]), seriousness [32,33] and expectedness [32,33]. With regards to seriousness, ADRs were considered serious if they resulted in one of the following outcomes: death, life-threatening condition, inpatient hospitalization, or prolongation of existing hospitalization, persistent or significant disability/incapacity, a congenital anomaly/birth defect or another important medical event. Additionally, in respect of expectedness, ADRs were considered unexpected if their nature of severity was not consistent with the applicable summary of product characteristics.

### 2.6. Statistical Analysis

Statistical analyses were performed by using the statistical program IBM SPSS Statistics version 25 (IBM, Armonk, NY, USA) applying a significance level of 0.05. Descriptive statistics were used to present the general characteristics of the respondents, and the collected data were presented using frequency, percentage, mean and standard deviation, median and inter-quartile range. To test the normality of the data distribution, the Kolmogorov–Smirnov test was used. The Wilcoxon signed-rank test was used to analyze the change from baseline to end-point values of the EQ-5D-5L and VAS scale. T-test was applied to determine the difference between baseline and end-point rates of suspected ADR reports, while the correlation between the rate of suspected ADR reports and number of drugs used, number of comorbidities and age was determined with Pearson’s correlation.

## 3. Results

During the study period, a total of 69 patients were enrolled. Following a dropout rate of 5.8 % (one patient died and three patients dropped out of the study after losing interest in further participation), 65 participants (22 men and 43 women) aged 72.4 ± 4.6 years (mean ± SD) completed the study. Detailed participant characteristics that include sociodemographic and clinical data are depicted in Table 1.

### 3.1. Health-Related Quality of Life

The health profiles of patients based on their answers to the EQ-5D-5L questionnaire are depicted in Table 2. At the end of the study, none of the patients stated to have an extreme level in any EQ-5D dimension. Overall, following the intervention, patients reported a significant improvement in dimensions “self-care” (*p* = 0.011) and “usual activities” (*p* = 0.003), whereas in the “mobility” (*p* = 0.203), “pain/discomfort” (*p* = 0.173) and “anxiety/depression” (*p* = 0.083) dimensions, no significant change was found. Results obtained using the self-rated VAS scale demonstrate that the CMM services had no impact on the self-assessed health (*p* = 0.781), with a mean value of 57.42 at the baseline and 57.67 at the end of the study.

### 3.2. Adverse Drug Reactions

**Figure 1:**
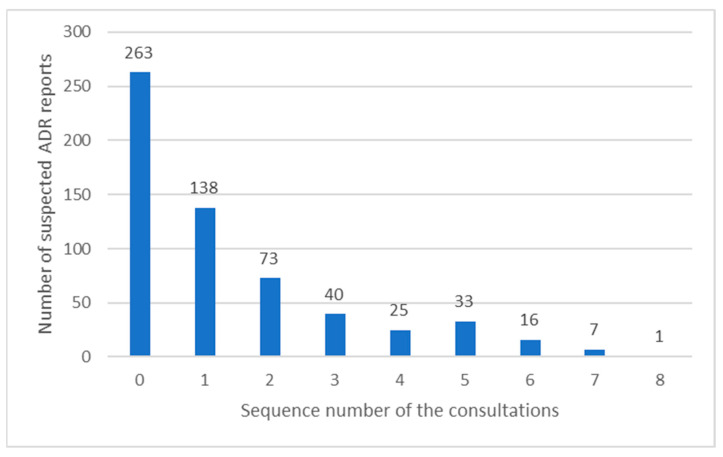
The prevalence of suspected ADRs reported according to the sequence number of consultations patients attended.

Altogether, a total of 596 suspected ADR reports were found, with a mean ± SD of 9.2 ± 16.9 per patient, out of which 67.3% were experienced by patients prior to undergoing CMM services and were reported at the initial assessment. Surprisingly, only one patient did not experience any ADR, whereas one patient experienced as many as 138 ADRs. The majority of reported ADRs concerned women (77.3%), with a mean value of ADRs being 10.6 +/− 20.7, as opposed to 6.8 +/− 4.5 in men. There was a strong, positive correlation between the number of drugs used and the rate of suspected ADRs (r = 0.823, *p* < 0.001). However, the correlation between the older age and the number of comorbidities and a higher rate of ADRs, previously reported in the literature, was not found in our study. The rate of suspected ADRs declined with the number of consultations patients attended (Figure 1). Therewithal, a positive and statistically significant impact of CMM services on the reduction in the rate of suspected ADRs was observed, namely 2.7 ± 1.7 ADRs per patient at the initial assessment vs. 1.0 ± 1.5 ADRs per patient at the last consultation (*p* < 0.001).

Reported suspected ADRs were further classified according to MedDRA SOC, occurrence mechanism (by Edwards and Aronson), seriousness and expectedness, as shown in Table 3.

## 4. Discussion

CMM, as found in our study, is a large-scale intervention shown to have a beneficial impact on patients’ HRQoL. To our knowledge, this is the first prospective study evaluating the impact of CMM services on HRQoL, regardless of the patient sample, clinical setting or instrument used. Epidemiological data confirm that CVDs are the leading cause of death, taking yearly an estimated 17.9 million lives, with Croatia being no exception [1,10]. Hence, the CMM services employed in our study targeted patients with CVDs as these are among the most prevalent and costly chronic diseases worldwide.

Health-related quality of life, alongside clinical outcome measures, is a crucial outcome in patients with chronic diseases since, in some instances, the value of a particular intervention can only be described by the patient. The EQ-5D-5L instrument used to evaluate the HRQoL in our study is a generic patient-reported outcome measure (PROM) used to assess a patient’s health status at a particular point in time. PROMs present an important part of the patient-centered approach as they are measured from the patient’s viewpoint and are used to more fully evaluate the quality of care [34]. Clearly, HRQoL represents an important indicator of the benefit pharmaceuticals and pharmacy interventions offer, and although still underused, it is likely to increase over time as it can be employed by various stakeholders in the decision-making process.

The obtained results indicate that CMM services have a positive impact on two dimensions of patients’ HRQoL, “self-care” and “usual activities”, with no significant impact on the remaining three dimensions, hence rendering the overall EQ-5D-5L health status improved [26]. Namely, an EQ-5D health state was deemed to be “better” over time if it was better in at least one dimension and no worse in any other dimension [27]. Moreover, our study incorporated multiple in-person and online consultations over a one-year period and was partially conducted during the COVID-19 lockdown. Patients were at home most of the time, and therefore, their mobility was indeed limited to the in-house setting. In spite of that, we did not find any deterioration in the “mobility” dimension nor in the “anxiety/depression” dimension, both of which were seriously affected by the pandemic. The fact that patients neither reported nor perceived their physical and/or psychological status worsened, but rather comparable, is considered a favorable result, given that the COVID-19 pandemic did not leave any sphere of life or public health system intact. That said, CMM appears to be a good solution for addressing non-optimal medication management as it improved patients’ HRQoL and as such should be considered for implementation in the healthcare system.

Moreover, analysis of the EQ-VAS scale, which represents patients’ perspective, did not reveal any significant change between the two time points. This result is in accordance with other studies that have looked at the impact of various pharmaceutical care interventions on a specter of diseases and have not found any significant improvement in the EQ-VAS score [35,36,37]. It could be argued that the VAS score is not strictly defined as the abovementioned EQ-5D-5L dimensions, allowing every patient to perceive the scale differently. Interestingly, 26% of patients at baseline and 28% after the 12-month follow-up marked their health in the middle (at exactly 50), and this preference was also shown in other studies [37]. There is a possibility that patients with chronic diseases had already got used to their health conditions [38], and therefore, they chose a score exactly in-between the two extremes.

Various studies have investigated the influence of a multitude of pharmacy interventions on patients’ HRQoL and have found diverse results [39]. Namely, in addition to the lack of pharmaceutical care particular measures for HRQoL, the lack of standardization in the reporting of pharmaceutical care interventions [40] and the heterogeneity of the services provided might be responsible for the variability in the pharmaceutical care impact on HRQoL outcomes. Moreover, majority of the studies that have used the EQ-5D-5L as an assessment tool [35,36,41,42,43,44,45,46,47] have not found any significant impact of the pharmacists’ intervention on HRQoL, irrespective of the clinical setting or study design. Statistically significant HRQoL between-group differences were observed in a study that aimed to determine the impact of a community pharmacist’s intervention on patients who had initiated antidepressant treatment, indicating that patients who received extra pharmaceutical care perceived improved HRQoL [48]. The authors challenged their results by stating that the effect size was small to moderate, making the clinical relevance of this difference questionable.

Additionally, to the best of our knowledge, this is the first study to have analyzed the impact of CMM services on the prevalence of adverse drug reactions. Despite ADRs being one of the main causes of morbidity and mortality worldwide [49,50], thus adversely influencing the clinical outcomes and quality of life as well as burdening limited health care budgets [51], only a small body of literature has thus far analyzed the epidemiology of ADRs in the primary care setting. The currently available body of literature unambiguously shows rather wide prevalence rates of reported ADRs, from only 6% to as much as 80% [23,52], largely due to the fact that as many as 95% of all ADRs are not even being reported [53]. Notably, the higher prevalence of suspected ADRs reported in this study can indubitably be explained by the comprehensive data collection process conducted within the CMM services as well as by the patients’ characteristics and prospective study design. Every consultation begins with uncovering patients’ medication experience followed by a detailed assessment of patients’ medication history and current medical record. As such, CMM contributes unique data and valuable new knowledge on the effectiveness and safety of medications in practice [54] and, as found in our study, reduces the prevalence of ADRs.

In this study, based on the occurrence mechanism, type A ADRs made up the majority of the total number of reported ADRs. On account of being a result of an exaggeration of a drug’s pharmacological effect, type A ADRs are predictable and as such potentially or definitely avoidable. Notwithstanding the relatively small patient sample included in this study for a limited period of time, 73 serious and 106 unexpected ADRs were reported (12.3% and 18.5%, respectively). These findings denote that there is plenty of room for improvement in the care of elderly cardiovascular patients. Taking into consideration the increasing number of medications patients take, the ever more complex therapy regimens and the increasing number of healthcare professionals that can prescribe medications, on the one hand, and the lack of control over the prescription and consumption of medications, on the other [9,55,56], CMM services could serve as a solution to the evergrowing clinical, financial and humanistic burden of ADRs through more careful selection and more frequent monitoring of patients’ therapy. Furthermore, an extremely high prevalence of ADRs reported at patients’ initial assessment in this study must have contributed to the poor baseline patients’ quality of life pointing to the fact that a greater emphasis should be put on measuring the quality of life in patients with ADRs.

This study had a number of limitations. First, it was conducted on a relatively small patient sample and in only one health center, thus limiting the generalizability of study results. Second, the lack of a control group could have led to the misinterpretation of the obtained results as it is harder to be certain that the outcome was caused by the experimental treatment or new service and not by other variables. Third, it was recently found that HRQoL measures used in pharmaceutical care studies provide very limited coverage of themes related to the burden of medicine on HRQoL and may have limited potential for use as a sole humanistic measure when evaluating pharmaceutical care interventions [15]. Fourth, patients did not fill out the questionnaire completely on their own but with a help of a pharmacist-researcher which could have inadvertently influenced the patients’ responses, leading to the introduction of bias. On the other hand, this can be regarded as a strength of the study, especially since the patients were of older age and had some limitations in understanding and reading the questionnaire. Hence, they were assisted by a pharmacist-researcher who could have addressed patients’ questions and clarified the meaning of particular dimensions of the EQ-5D-5L. Finally, it should be noted that one patient experienced a significant proportion of ADRs recorded by the study, potentially compromising the data analysis as an outlier.

## 5. Conclusions

In conclusion, the results of the present study indicate that comprehensive medication management services provided at the primary care level may improve health-related quality of life in older patients with CVDs. Furthermore, CMM services detected a large amount of ADRs and significantly diminished the proportion of ADRs following 1-year patient follow-up rendering this pharmacist-led intervention a viable solution for safety management. Considering the fact that CMM improved patients’ HRQoL and patients’ well-being along with patient safety, it should be considered for implementation in the healthcare system as an effective solution for addressing medication mismanagement and irrational drug use.

## Figures and Tables

**Table 1:** Patient population receiving comprehensive medication management services.

Patient Characteristic	Group
	Intervention
Sample size (*n*)	65
Age (years) *	72.4 ± 4.6
Sex female male	43 22
Body mass index	29.5 ± 4.9
Alcohol consumption yes/no	15/50
Cigarette consumption yes/no	2/63
Status of physical activity yes/no	28/37
Level of education primary/secondary/higher	3/31/29
≥5 medications used (polypharmacy) yes/no	64/1
Patients diagnosed with type 2 diabetes mellitus yes/no	26/39
Patients diagnosed with hyperlipidemia yes/no	35/30
Number of medications per patient at the initial visit *	10.8 ± 3.6
Number of medications used at the initial visit	699
Use of cardiovascular system medications, *n* (%)	267 (38.2)
Use of gastrointestinal system and endocrine system medications, *n* (%)	123 (17.6)
Use of nervous system medications, *n* (%)	78 (11.2)
Number of diagnoses per patient at the initial visit *	7.9 ± 3.4
Number of diagnoses	510
Diseases of the circulatory system, %	32.5
Endocrine, nutritional and metabolic diseases, %	21.6
Diseases of the musculoskeletal system and connective tissue, %	11.2

* Data expressed as mean ± SD.

**Table 2:** Health state profiles according to the EQ-5D-5L dimensions.

EQ-5D-5L Dimension		T0 * (%) *n* = 60	T1 * (%) *n* = 58	*p* Value
MOBILITY	No problems	38.3	37.9	
	Slight problems	18.3	32.8	
	Moderate problems	28.3	19.0	0.203
	Severe problems	15.0	10.3	
	Unable to walk	0.0	0.0	
SELF-CARE	No problems	78.3	89.7	
	Slight problems	10.0	6.9	
	Moderate problems	6.7	1.7	0.011
	Severe problems	3.3	1.7	
	Unable to do	1.7	0.0	
USUAL ACTIVITIES	No problems	50.0	63.8	
	Slight problems	26.7	20.7	
	Moderate problems	16.7	12.1	0.003
	Severe problems	3.3	3.4	
	Unable to do	3.3	0.0	
PAIN/DISCOMFORT	No pain	16.7	20.7	
	Slight pain	26.7	29.3	
	Moderate pain	31.7	34.5	0.173
	Severe pain	23.3	15.5	
	Extreme pain	1.7	0.0	
ANXIETY/ DEPRESSION	Not anxious or depressed	51.7	39.7	
	Slightly anxious or depressed	31.7	31.0	
	Moderately anxious or depressed	11.7	20.7	0.083
	Severely anxious or depressed	5.0	8.6	
	Extremely anxious or depressed	0.0	0.0	

* T0, baseline; T1, after 12 months.

**Table 3:** Distribution of reported suspected ADRs classified by MedDRA SOC, occurrence mechanism (by Edwards and Aronson), seriousness and expectedness.

Classification	*n* (%) of Suspected ADRs
**MedDRA SOC [31]**	
General disorders and administration site conditions	103 (17.28%)
Vascular disorders	71 (11.91%)
Gastrointestinal disorders	68 (11.41%)
Musculoskeletal and connective tissue disorders	61 (10.23%)
Nervous system disorders	59 (9.90%)
Skin and subcutaneous tissue disorders	37 (6.21%)
Renal and urinary disorders	37 (6.21%)
Metabolism and nutrition disorders	32 (5.37%)
Cardiac disorders	26 (4.36%)
Respiratory, thoracic and mediastinal disorders	20 (3.36%)
Investigations	19 (3.19%)
Ear and labyrinth disorders	17 (2.85%)
Psychiatric disorders	11 (1.85%)
Immune system disorders	10 (1.68%)
Reproductive system and breast disorders	9 (1.51%)
Eye disorders	7 (1.17%)
Endocrine disorders	6 (1.01%)
Infections and infestations	2 (0.34%)
Injury, poisoning and procedural complications	1 (0.17%)
Occurrence mechanism (by Edwards and Aronson) *	
Type A	448 (75.17%)
Type B	110 (18.46%)
Type C	9 (1.51%)
Type D	29 (4.87%)
**Seriousness**	
Serious	73 (12.25%)
Non-serious	523 (87.75%)
**Expectedness**	
Expected	465 (81.44%)
Unexpected	106 (18.56%)

ADR, adverse drug reaction. MedDRA SOC, Medical Dictionary for Regulatory Activities System Organ Class. * Occurrence mechanism (**by Edwards and Aronson**)—ADRs classified into six types: dose-related (A), non-dose-related (B), dose-related and time-related (C), time-related (D), withdrawal (E) and failure of therapy (F).

## Data Availability

The datasets generated during and/or analyzed during the current study are available from the corresponding author upon reasonable request.
